# mtDNA as a Mediator for Expression of Hypoxia-Inducible Factor 1α and ROS in Hypoxic Neuroblastoma Cells

**DOI:** 10.3390/ijms18061220

**Published:** 2017-06-07

**Authors:** Chung-Wen Kuo, Meng-Han Tsai, Tsu-Kung Lin, Mao-Meng Tiao, Pei-Wen Wang, Jiin-Haur Chuang, Shang-Der Chen, Chia-Wei Liou

**Affiliations:** 1 Department of Neurology, Kaohsiung Chang Gung Memorial Hospital and Chang Gung University College of Medicine, Kaohsiung 833, Taiwan; bulakuo@gmail.com (C.-W.K.); menghan@cgmh.org.tw (M.-H.T.); tklin@adm.cgmh.org.tw (T.-K.L.); jp1916@ms4.hinet.net (S.-D.C.); 2 Department of Medical Research, Kaohsiung Chang Gung Memorial Hospital, Kaohsiung 833, Taiwan; 3 Departments of Pediatrics, Kaohsiung Chang Gung Memorial Hospital and Chang Gung University College of Medicine, Kaohsiung 833, Taiwan; tmm@adm.cgmh.org.tw; 4 Department of Metabolism, Kaohsiung Chang Gung Memorial Hospital and Chang Gung University College of Medicine, Kaohsiung 833, Taiwan; WangPW@adm.cgmh.org.tw; 5 Departments of Pediatrics Surgery, Kaohsiung Chang Gung Memorial Hospital and Chang Gung University College of Medicine, Kaohsiung 833, Taiwan; jhchuang@adm.cgmh.org.tw; 6 Mitochondrial Research Unit, Kaohsiung Chang Gung Memorial Hospital and Chang Gung University College of Medicine, Kaohsiung 833, Taiwan

**Keywords:** hypoxia, HIF-1α, DRP1, ROS, mtDNA

## Abstract

Mitochondria consume O_2_ to produce ATP and are critical for adaption of hypoxia, but the role of mitochondria in HIF-1α pathway is as yet unclear. In this study, mitochondrial DNA (mtDNA) enriched (SK-N-AS) and depleted (ρ^0^) cells of neuroblastoma were cultured in a hypoxic chamber to simulate a hypoxic condition and then the major components involved in mitochondrial related pathways, hypoxia-inducible factor 1α (HIF-1α) and reactive oxygen species (ROS) were measured. The results showed that hypoxia-stimulated exposure elevated expression of HIF-1α, which was additionally influenced by level of generated ROS within the cytosol. Moreover, elevation of HIF-1α also resulted in increases of lactate dehydrogenase A (LDH-A) and pyruvate dehydrogenase kinase 1 (PDK1) in both hypoxic cells. The expression of mitochondrial biogenesis related proteins and metabolic components were noted to increase significantly in hypoxic SK-N-AS cells, indicating that mtDNA was involved in mitochondrial retrograde signaling and metabolic pathways. An analysis of dynamic proteins found elevated levels of HIF-1α causing an increased expression of dynamin-related protein 1 (DRP1) during hypoxia; further, the existence of mtDNA also resulted in higher expression of DRP1 during hypoxia. By using siRNA of HIF-1α or DRP1, expression of DRP1 decreased after suppression of HIF-1α; moreover, the expression of HIF-1α was also affected by the suppression of DRP1. In this study, we demonstrated that mtDNA is a mediator of HIF-1α in eliciting metabolic reprogramming, and mitochondrial biogenesis. Identification of a mutual relationship between HIF-1α and DRP1 may be a critical tool in the future development of clinical applications.

## 1. Introduction

Hypoxia-inducible factor 1α (HIF-1α) is a DNA binding transcription factor activated by compromised oxygen tension. It promotes the up-regulation of genes that control cellular adaptive responses and was found to have a role on hypoxic-ischemia events, including limb ischemia and myocardial infarction [1,2,3,4]. In the central nervous system, hypoxia could cause devastating brain diseases, such as ischemic stroke and hypoxic encephalopathy, which frequently results in significant disability and burden for the health care system. A therapeutic approach to increase the survival of brain cells during a low oxygen situation is crucial to improve long-term outcomes. Therefore, the HIF-1α pathway could be a potential target for development of novel therapies for ischemic stroke. Hitherto, only a few studies have investigated the role of HIF-1α in neuronal tissues or animal models of ischemic stroke and demonstrated potential benefits of such therapy [5,6,7,8,9]. Mitochondrion is the key organelle responsible for energy production and oxygen consumption. Reactive oxygen species (ROS), derived from mitochondria, have been demonstrated to regulate the induction and hypoxic activation of HIF-1α [10]. However, more recent studies have yielded controversial results [11,12,13]. One explanation is that mitochondrial ROS generation may be cell-type specific [13]. Other studies have shown that mitochondria respond to environmental change through several different mechanisms, including biogenesis and expression of dynamic proteins that regulate mitochondrial fission and fusion [14,15,16]. Mitochondria respond to a hypoxic environment through a reprogramming of the energy supply: shifting from aerobic to anaerobic pathway for energy production. Furthermore, mitochondria can also undergo constant fission and fusion to repair damage component of mitochondria. The mechanism allows for segregation of damaged mitochondria via fission, and gains material from healthy mitochondria via fusion. This dynamic process is regulated by a number of proteins: mitofusin 1 (MFN1), mitofusin 2 (MFN2), dynamin-related protein 1 (DRP1), human fission factor-1 (FIS1) and optic atrophy factor 1 (OPA1) [14,15,16]. HIF-1α has been suggested to mediate these adaptive responses.

To evaluate the complex relationship among ROS, HIF-1α and mitochondrial adaptation ability under hypoxic conditions, we investigated here the generation of ROS, expression of HIF-1α, morphogenetic proteins, and cellular energetic biomarkers by using central nervous system derived SK-N-AS human neuroblastoma cell line. We further create mitochondrial DNA-depleted SK-N-AS cells (ρ^0^ cells) and use siRNA of HIF-1α to clarify the role of mitochondrial DNA (mtDNA) and HIF-1α in the hypoxic adaptive response, respectively. According to these data, we will evaluate the role of mtDNA in hypoxic SK-N-AS cells.

## 2. Results

### 2.1. The Expression of HIF-1α in SK-N-AS and ρ^0^ Cells under Hypoxia

**Figure 1:**
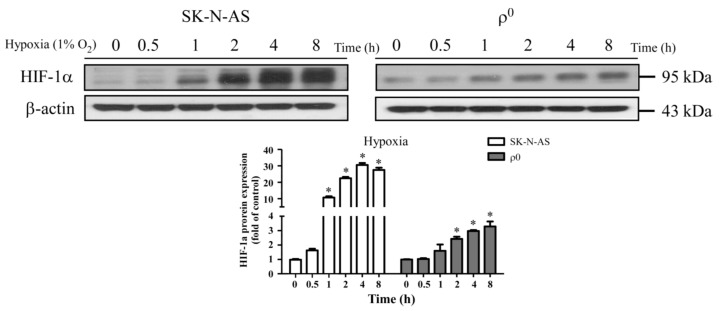
Hypoxia induced factor 1α (HIF-1α) was activated in hypoxic SK-N-AS and ρ^0^ cells. The upper panel shows HIF-1α protein expression with immunoblotting after different hypoxic times in SK-N-AS and ρ^0^ cells; the bar chart below illustrates HIF-1α protein expression change with different hypoxic times (relative quantification to β-actin). Data are shown as mean ± SD of three independent experiments. * Represented *p* < 0.05 when compared to normoxic condition was determined using one-way ANOVA.

To study whether HIF-1α expressed in hypoxic cells, SK-N-AS and ρ^0^ cells were both treated with different hypoxic times, and then the expression of HIF-1α protein were determined by Western blot. The result showed that HIF-1α persistent expressed in both cells with longer hypoxic times (Figure 1A). Furthermore, the expression level of HIF-1α in hypoxic SK-N-AS cells was more than hypoxic ρ^0^ cells. The period of four hours of hypoxia exposure was selected due to the highest induction of HIF-1α, but only mild degree of decreased viability in both cellular lines (Figure S1A). We also further conducted a study of the functional differences between the two cell lines, and the results show no significant differences in cellular viability or ATP production between SK-N-AS and ρ^0^ cells after hypoxia exposure for four hours (Figure S1B). Moreover, SK-N-AS and ρ^0^ cells were cultured under 1% and 21% oxygen concentration for 4 h and prepared for determining immunofluorescence and mRNA expression of HIF-1α. The results showed that expressions of HIF-1α were both significantly increased in SK-N-AS and ρ^0^ cells under 1% compared to 21% oxygen exposure (Figure S2A), while this phenomenon remains consistent for the mRNA expression of HIF-1α (Figure S2B).

### 2.2. The Changes of ROS and Mitochondrial Membrane Potential under Hypoxic

**Figure 2:**
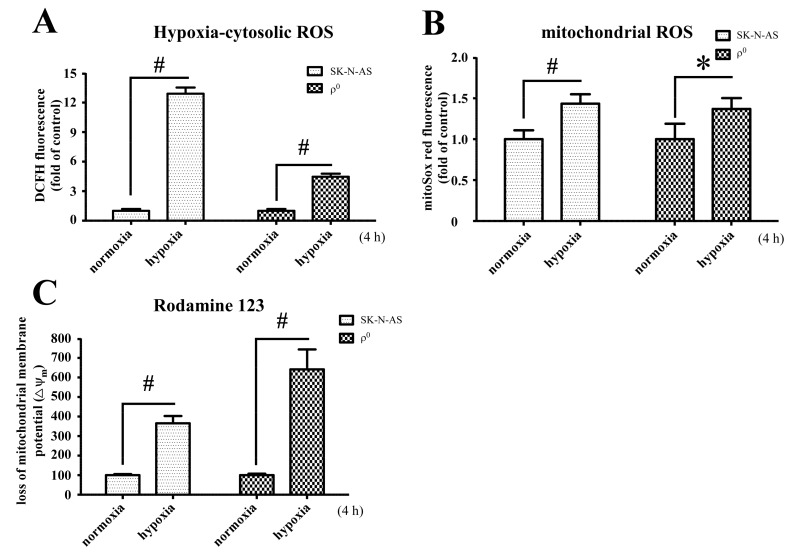
Hypoxia and mitochondrial DNA (mtDNA) affect the production of ROS and loss of mitochondrial membrane potential (Δψ_m_). Intracellular H_2_O_2_ production (**A**); or mitochondrial superoxide (**B**) under normoxia or after hypoxia for 4 h were determined by flow cytometry with DCFH-DA or MitoSox red individually in both SK-N-AS and ρ^0^ cells; (**C**) Loss of mitochondrial membrane potential in normoxic or hypoxic SK-N-AS and ρ^0^ cells were measured by Rodamine 123. The percentages loss of Δψm indicated the number of Δψm collapsed cells after exposure to hypoxia. These results are shown as mean ± SD of more than three independent experiments. Statistical significance (* *p* < 0.05, # *p* < 0.005) was determined using the one-way ANOVA compared to normoxia.

To further investigate the ROS and membrane potential in hypoxic SK-N-AS and ρ^0^ cells, cells were treated with 21% or 1% O_2_ for 4 h and then incubated with DCFH-DA and MitoSox Red. The results showed that the amounts of intracellular ROS generation after hypoxia was significantly increased in both SK-N-AS and ρ^0^ cells, although tend to be lesser increase in ρ^0^ cells (Figure 2A). However, mitochondrial ROS was slightly increased in both cells after hypoxic exposure (Figure 2B). Besides, the elevated levels of cytosolic ROS respond to hypoxia in both cells were higher than mitochondrial ROS (Figure 2A,B). In addition, hypoxia also caused significant loss of mitochondrial membrane potential measured by Rodamine 123, especially more prominent in ρ^0^ cells (Figure 2C). To compare relative levels of ROS and membrane potential between the two cell lines, we also adjusted accordingly by using normoxic SK-N-AS group to normalize the other groups. The results showed that cellular ROS in both hypoxic cells were higher than normoxic SK-N-AS cells (Figure S3A), but this phenomenon was not observed in mitochondrial ROS (Figure S3B). Furthermore, there was no significant different in lsot of membrane potential in both hypoxic cells if normalized with normoxic SK-N-AS cells (Figure S3C).

### 2.3. The Expression of Metabolism Related Proteins in Hypoxia

**Figure 3:**
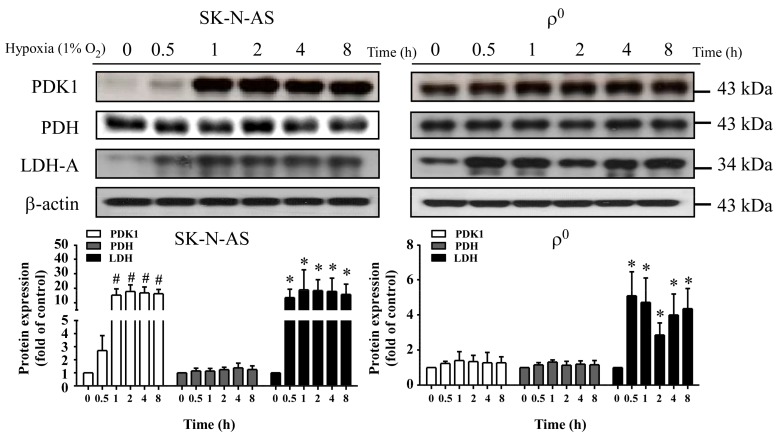
Effect of hypoxia on metabolism-related proteins in SK-N-AS and 2137 ρ^0^ cells. Upper panel shows immunoblotting of the expression of pyruvate dehydrogenase kinase 1 (PDK1), pyruvate dehydrogenase (PDH) and lactate dehydrogenase A (LDH-A) proteins after different hypoxic times; the bar chart in lower panel shows the relative changes (to β-actin) of PDK1, PDH and LDH-A proteins. The results are shown as mean ± SD of more than three separate experiments in which similar results were obtained. * Represented *p* < 0.05 and # represented *p* < 0.005 when compared to normoxic condition were determined using the one-way ANOVA.

To study the relationship between hypoxia and metabolic flux, we detected the expression of metabolism related proteins in hypoxic cells by using Western blot. In the baseline situation, high lactate dehydrogenase A (LDH-A) combined with high pyruvate dehydrogenase kinase 1 (PDK1) was noted in mtDNA-depleted cells which reflects their sustaining of energy supply through anaerobic respiration. Whereas, a high pyruvate dehydrogenase (PDH) but low LDH-A and PDK1 was noted in mtDNA-enriched cells, which is appropriate for their supply of energy through an aerobic respiration pathway (Figure 3). After exposure to hypoxic condition in SK-N-AS cells, expression level of LDH-A was significantly elevated to 20 folds compared to baseline (Figure 3, left panel). This phenomenon was also discovered in ρ^0^ cells, although not as strong as in SK-N-AS cells (Figure 3, right panel). Moreover, the expressions of PDK1 were also elevated in both hypoxic cells (Figure 3). However, the expression levels of PDH were not altered in both hypoxic cells (Figure 3).

### 2.4. Hypoxia Affects Mitochondrial Biogenesis

**Figure 4:**
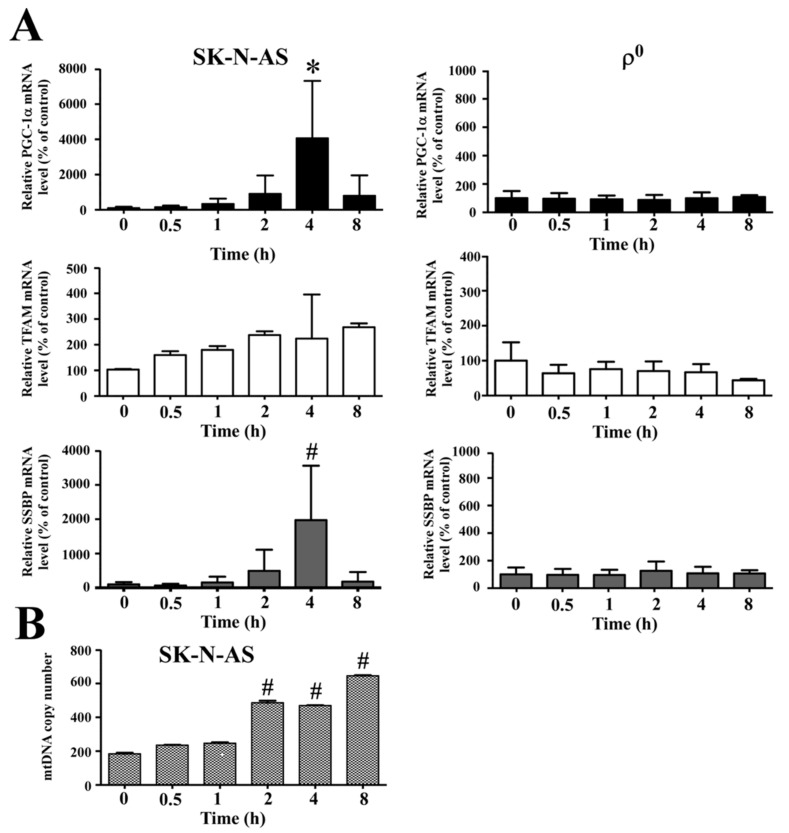
Expression of mitochondrial biogenesis-related genes after hypoxia. (**A**) The mRNA expression level of peroxisome proliferation-activated receptor γ coactivator-1 α (PGC-1α), mitochondrial transcription factor A (TFAM) and single strand DNA binding protein (SSBP) after different hypoxic times in SK-N-AS (left panel) or ρ^0^ (right panel) cells were determined by q-PCR; (**B**) Mitochondrial DNA copy number changes in SK-N-AS cells after different hypoxic times were also measured by q-PCR. Data are shown as mean ± SD of three separate experiments. * Represented *p* < 0.05 and # represented *p* < 0.005 when compared to normoxic condition were determined using the one-way ANOVA.

Previous study had shown that hypoxia induced expression of peroxisome proliferation-activated receptor γ coactivator-1 α (PGC-1α) and mitochondrial biogenesis in the myocardium. Because PGC-1α is a transcriptional factor of mitochondrial biogenesis, it regulates expression of mitochondrial transcription factor A (TFAM) to activate mitochondrial biogenesis. To rule out how hypoxia affects mitochondrial biogenesis in SK-N-AS cells, we detected the expressions of mRNA which were mitochondrial biogenesis related gene by using q-PCR. The result showed that mRNA levels of PGC-1α, TFAM and single strand DNA binding protein (SSBP) genes were all up-regulated in SK-N-AS cells in response to hypoxia, while the same response was absent in ρ^0^ cells (Figure 4A). Since hypoxia enhanced the expression of mitochondrial biogenesis related gene, we further detected the mtDNA copy numbers by using q-PCR. The result showed that mtDNA copy numbers were indeed increased in SK-N-AS cells after exposing different hypoxic times (Figure 4B).

### 2.5. Mitochondrial Dynamics in Hypoxic Cells

**Figure 5:**
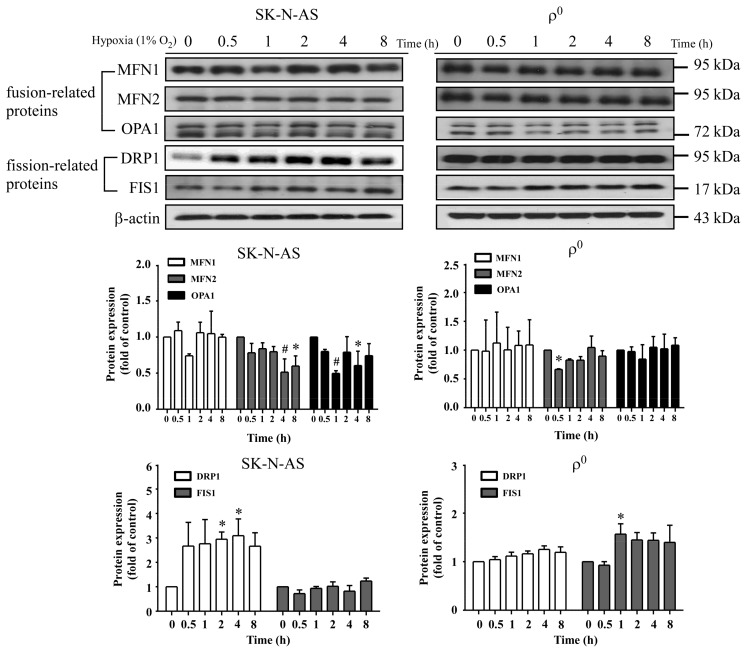
The effect of hypoxia on mitochondrial dynamic related proteins. The representative Western blot and the quantification analysis of mitofusin 1 (MFN1), mitofusin 2 (MFN2), optic atrophy factor 1 (OPA1), dynamin-related protein 1 (DRP1) and human fission factor-1 (FIS1) were determined in SK-N-AS (left panel) or ρ^0^ (right panel) cells after different hypoxic times exposure. Data are shown as mean ± SD of three separate experiments. * Represented *p* < 0.05 and # represented *p* < 0.005 when compared to 21% O_2_ baseline were determined using the one-way ANOVA.

**Figure 6:**
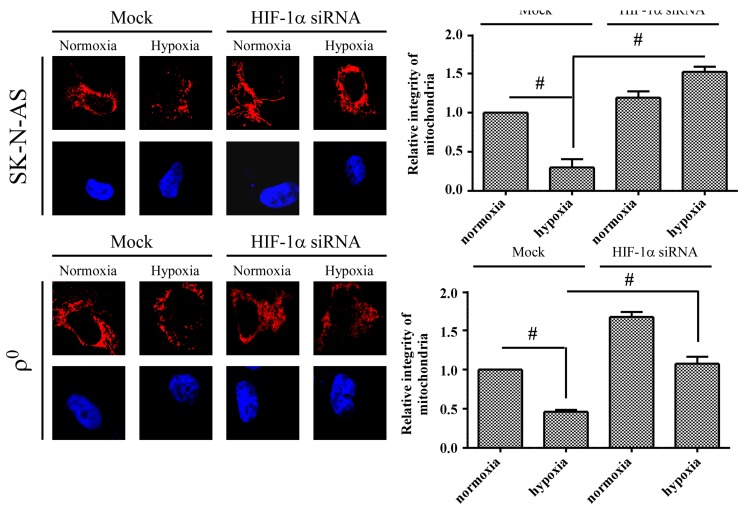
The effect of hypoxia and HIF-1α on mitochondrial morphology. After transfecting with 20 nM non-targeting siRNA (Mock) or HIF-1α siRNA (right panel) for 24 h, neuroblastoma cells were further treated with 4 h of hypoxic condition. Morphology of mitochondria in neuroblastoma cells were stained with MitoSOX Red (0.5 µg) and nuclei were stained with DAPI showing blue fluorescent. Graphs show the relative integrity area of mitochondria by using wand tool function of software ImageJ. Data are shown as mean ± SD of three separate experiments. # Represented *p* < 0.005 when compared to normoxic Mock condition was determined using the *t*-Test.

Because HIF-1α has also been reported to regulate mitochondrial fission, we further detected the expression of mitochondrial fusion-related (MFN1, MFN2 and OPA1) or fission-related (DRP1 and FIS1) proteins after hypoxia. The result showed that only DRP1 protein expression was significantly increased in SK-N-AS cells, and this response was not seen in ρ^0^ cells (Figure 5). DRP1 is a member of the dynamin family of large GTPase and it controls the final part of mitochondrial fission. Our data demonstrated that DRP1 protein increased in hypoxic cell, therefore, we further observe the morphology of mitochondria after hypoxia under fluorescent microscope. The results showed that SK-N-AS and ρ^0^ cells both contained fragmentation of mitochondrion after hypoxic treatment (Figure 6). Moreover, we also transfected SK-N-AS and ρ^0^ cells with non-targeting siRNA or HIF-1α siRNA, and then treated these cells with or without hypoxia. The fluorescence results showed that HIF-1α siRNA reduced mitochondrial fission especially in SK-N-AS cells (Figure 6). Furthermore, the relative integrity of mitochondrial area in fluorescent results were determined by ImageJ. The results showed that integrity of mitochondria decreased after hypoxia, but increased with transfecting HIF-1α siRNA in hypoxic SK-N-AS cells (Figure 6), and this phenomenon was also observed in ρ^0^ cells (Figure 6).

### 2.6. The Effect of HIF-1α and DRP1 siRNA

**Figure 7:**
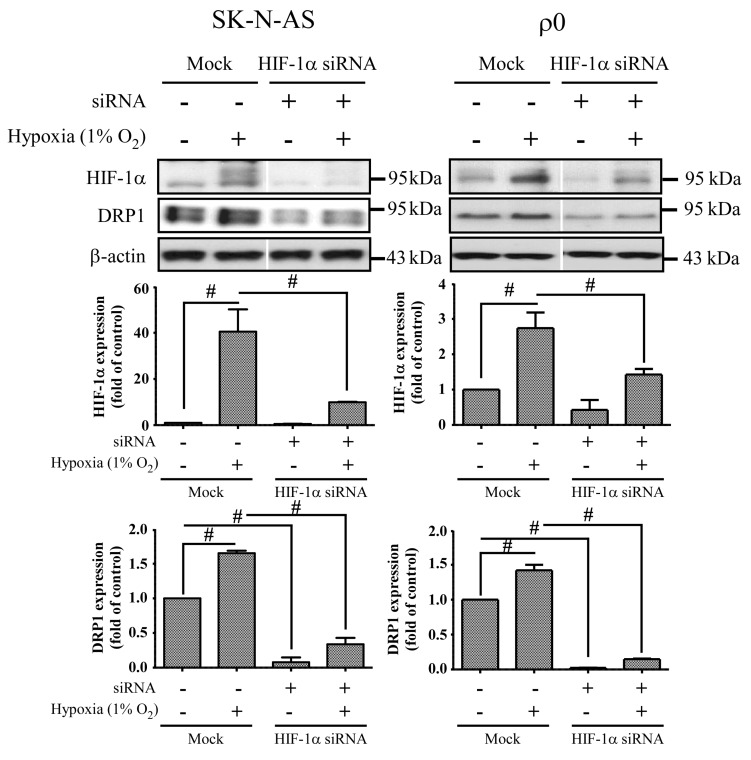
The effect of HIF-1α siRNA. Cells were transiently transfected with siRNA targeted for HIF-1α (HIF-1α siRNA) or non-specific siRNA (Mock) for 24 h, and then incubated in normoxia or 1% O_2_ for 4 h. The expression of HIF-1α, DRP1 and β-actin proteins was revealed by Western blot analysis, and the quantification analyses are shown below. β-Actin was used as a loading control. The results are shown as mean ± SD of at least three experiments. # Represented *p* < 0.005 when compared to normoxic Mock condition was determined using the one-way ANOVA.

**Figure 8:**
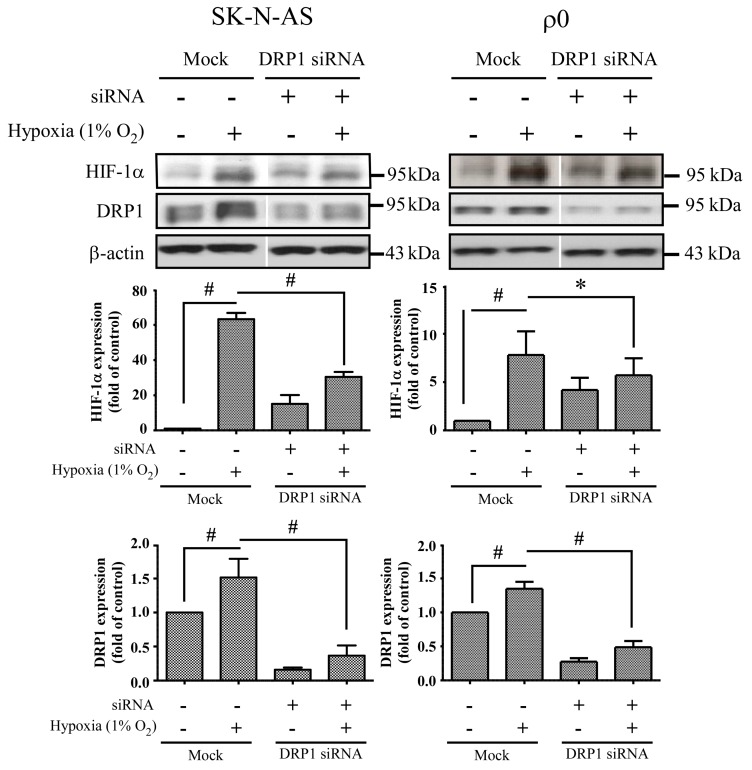
The effect of DRP1 siRNA. Cells were transiently transfected with siRNA targeted for DRP1 (DRP1 siRNA) or non-specific siRNA (Mock) for 24 h, and then incubated in normoxia or 1% O_2_ for 4 h. The expression of HIF-1α, DRP1 and β-actin proteins were revealed by Western blot analysis, and the quantification analyses are shown below. β-Actin was used as a loading control. The results are shown as mean ± SD of at least three experiments. * Represented *p* < 0.05 and # represented *p* < 0.005 when compared to normoxic Mock condition were determined using the one-way ANOVA.

In order to clarify the response of mitochondria-related pathways during hypoxia is mediated by HIF-1α or DRP1, we transfected siRNA to suppress the expression of HIF-1α and DRP1 independently. The expression levels of DRP1 were decreased in both SK-N-AS and ρ^0^ cells after suppression of HIF-1α irrespective of exposure to hypoxic condition or not (Figure 7). On the other hand, the expression of HIF-1α also was decreased in hypoxic cells after suppression of DRP1, but elevated HIF-1α expression was observed in normoxic cells (Figure 8).

## 3. Discussion

Mitochondrion is an important organelle involved in cellular metabolism and is a processor responsible for the majority of intracellular oxygen consumption. The fundamental role of which is to offer sufficient ATP for maintaining cellular survival. During hypoxic conditions, adaptations of metabolic pathways are triggered by expression of HIF-1α. We found that mitochondria-derived ROS is involved in hypoxic signal transduction through the activation of HIF-1α, and this phenomenon is consistent with a previous study [10]. This process is aimed at prolonging cellular endurance to hypoxia by enhancing the anaerobic glycolysis pathway while restricting the mitochondria-dependent aerobic pathway. In this study, we explored the interaction between mitochondria and HIF-1α, and found that although mtDNA is not a prerequisite factor for the induction of hypoxia-related HIF-1α expression, it plays a role in the maintenance of its expression. Moreover, we found that mitochondria-derived ROS is involved in hypoxic signal transduction through the activation of HIF-1α. This effect is likely due to the generation of ROS with the presence of mtDNA, as shown by disparities in the elevation of cytosolic ROS levels.

Mitochondrial DNA encoded respiratory chain proteins, i.e., complex III, are crucial in the generation of ROS, a key factor for the up-regulation of HIF-1α translation [10]. Our study indicates that the presence of intact mitochondria, including mtDNA, is required for more effective production of cellular ROS and persistent activation of the HIF-1α pathway. A recent study also demonstrates that HIF-1α plays a central role in cellular respiration during hypoxic stress via regulation of cytochrome c oxidase (complex IV) activities [17]. Therefore, there is a possibility that HIF-1α and ROS have a reciprocal relationship, strengthening each other during the early phases of hypoxia. Furthermore, p53 has been reported to promote ubiquitination and degradation of HIF-1α in partially mtDNA-depleted cells [18], this may be another reason that HIF-1α expression is higher in SK-N-AS than ρ^0^ cells.

During hypoxia, HIF-1α plays a critical role in inducing expression of LDH-A, which enhances anaerobic glycolysis; whereas, it acts to indirectly inhibit PDH by up-regulating PDK1. In this study, we further evaluated the downstream effect of HIF-1α and found that the metabolism-related proteins LDH-A and PDK1 were up-regulated, whether in mtDNA enriched or depleted hypoxic cells; furthermore, we observed no difference in the expression of PDH, this may be caused by that elevated expression of PDK1 inactivate PDH and result in the suppression of tricarboxylic acid cycle (TCA) cycle [19]. Moreover, we found mitochondrial biogenesis was also up-regulated after hypoxia, as shown by increased mRNA levels of *PGC-1α*, *TFAM* and *SSBP* genes. Therefore, mtDNA copy number was also elevated, but this phenomenon was non-existent in mtDNA-depleted cells. This result is identical to our previous work which demonstrated the copy number of mtDNA below 1 copy per cell; furthermore, we showed that the ρ^0^ cells completely lacked expression of mitochondrial protein COX2 [20,21]. Several studies have also shown that mitochondrial biogenesis is induced by intracellular oxidative stress [22,23], therefore, mtDNA-depleted cells producing less cellular ROS may reduce the expressions of PGC-1α, TFAM and SSBP.

In this study, we found an increased expression of mitochondrial fission in response to hypoxia via the induction of HIF-1α. Mitochondrial fission is an essential process for inducing the proliferation of mitochondria, facilitating apoptosis during periods of cellular stress, and inducing the process of mitophagy. Fission and fusion of mitochondria play critical roles in maintaining functional activity during cellular response to stress. Mitochondrial fission creates new mitochondria, but also contributes to quality control by removing damaged mitochondria and facilitating apoptosis during severe stress [24]. Mitochondrial fission creates smaller and more discrete mitochondria, which is more capable of producing ROS to facilitate mitophagy and cell proliferation. It is possible that mitochondrial fission is the prelude of hypoxia-induced neuronal death, or the rescue response to hypoxia-induced mitochondrial damage. Mitochondria increases fission after hypoxia to remove damaged or depolarized mitochondria through mitophagy. In this process, mitochondria may be able to survive and replenish through mitochondrial biogenesis after the hypoxic conditions as they are constantly undergoing processes of fusion and fission. Therefore, an increase in fission-related protein DRP1 during hypoxia would accompany decreased expression of fusion-related proteins. Consequently, the results showing that fusion-related proteins MFN2 and OPA1 decrease in hypoxia were in line with expectations. Our study also confirms that mitochondrial fission due to hypoxia is mediated through HIF-1α, as demonstrated by a reduction of mitochondrial fission upon suppression of HIF-1α expression by using HIF-1α siRNA. HIF-1α’s role in inducing mitochondrial fission is likely to involve its mutual regulation with the mitochondrial fission protein DRP1.

We also found that mitochondrial dynamic protein DRP1 was altered after hypoxia and mediated through HIF-1α. DRP1 is a cytosolic protein with GTPase activity, and it regulates the process of mitochondrial fission. Furthermore, we found that DRP1 was reduced but not FIS1 when HIF-1α was silenced by siRNA of HIF-1α in both hypoxic SK-N-AS and ρ^0^ cells. On the other hand, repression of DRP1 expression by siRNA of DRP1 also decreases HIF-1α expression. Because DRP1 binds to p53 and sequesters p53 in mitochondria [18], cytosolic p53 level was increased when DRP1 was repressed, upon which cellular p53 promoted degradation of HIF-1α. These findings support the notion that mitochondrial fission is the consequence of HIF-1α induction under hypoxic conditions. In addition, we observed that DRP1 expression was obviously increased in ρ^0^ cells treated with combination of transfection and hypoxia than solely hypoxia. Therefore, we speculated that transfection reagent or non-specific dsRNA may be another stress to ρ^0^ cells. It is essential to further our understanding of these downstream effects of HIF-1α as this will assist in the development of neuroprotective treatments for hypoxic brain disorders, such as ischemic stroke or hypoxic encephalopathy.

### Limitations of the Present Study

The process of mtDNA-depleted cell selection by using EtBr could possibly lead to nuclear DNA damage, although there is as yet no conclusive data reported on this. Therefore, to purify the mtDNA of SK-N-AS cells and transfer mtDNA back into ρ^0^ cells for documentation purposes may be required in future laboratory work. In addition, it is technically difficult to create mtDNA-depleted cells in primary neuronal cells; therefore, the results of this study may reveal the mechanisms in cancer cells’ response to hypoxia which may not accurately reflect those of normal neuronal cells. However, the fact that HIF-1α induction is seen in human neuroblastoma cells [25] and primary rat neurons [26] is sufficient to imply that this induction is likely to occur in normal human neurons.

## 4. Materials and Methods

### 4.1. Cell Lines and Cell Culture

The human neuroblastoma cells (SK-N-AS, American Type Culture Collection, Manassas, VA, USA) were cultured with Dulbecco’s modified Eagle’s medium (DMEM) containing 10% (*v*/*v*) heat-inactivated fetal bovine serum (FBS; Invitrogen, Carlsbad, CA, USA), and antibiotic-antimycotic (Invitrogen) in 5% CO_2_/95% O_2_ humidified incubator at 37 °C. The cells were isolated and then re-cultured at a 1:10 ratio when the cells grew to 60–80% of confluence and maintained in DMEM containing 10% FBS, 25 mM HEPES, 110 mg/mL pyruvate and 50 ng/mL uridine (Invitrogen, Carlsbad, CA, USA) in 5% CO_2_/95% O_2_ humidified incubator at 37 °C. Furthermore, cells grew in a medium with 50 ng/mL ethidium bromide (EtBr) for 3 months to deplete mtDNA. A single clone was selected by limit dilution. The mtDNA-depleted ρ^0^ cells were confirmed by detection of mtDNA copy number, expression of mtDNA-coded proteins and oxygen consumption in the absence of EtBr [20,21]. The ρ^0^ cells were subcultured at a 1:3 ratio when the cells grew to 60–80% of confluence.

### 4.2. Quantification of mtDNA Copy Number by Real Time Polymerase Chain Reaction (PCR)

The cells were plated at a density of 2 × 10^6^ cells per well in 6-cm plates (Nunc, Roskilde, Denmark). After treatment in hypoxic camber, mtDNA was extracted from cells using QIAamp DNA Mini Kit (QIAGEN, Hilden, Germany). The copy numbers of relative mtDNA were evaluated by real-time PCR and normalized by simultaneous measuring of the nuclear DNA. The forward and reverse primers complementary to the *β-actin* gene were 5′-TCACCCACACTGTGCCCATCTCGA-3′ and 5′-CAGCGGAACCGCTCATTGCCAATGG-3′, respectively. The forward and reverse primers complementary to the sequence of the *ND2* gene of mtNDA were 5′-CACAGAAGCTGCCATCAAGTA-3′ and 5′-CCGGAGAG TATATTGTTGAAGAG-3′, respectively. The polymerase chain reaction (PCR) was performed by using the SYBR^®^ Green PCR Master Mix Kit (2×) (Applied Biosystems, Sparta Township, NJ, USA) in a LightCycler^®^ 480 System (Roche Applied Science, Mannheim, Germany). Ten nanograms DNA was mixed with 10 µL SYBR^®^ Green PCR Master Mix Kit with 500 nM of two primers to a final volume of 20 µL. The conditions of PCR were 10 min at 95 °C, followed by 45 cycles of denaturation at 95 °C for 10 s, annealing at 60 °C for 15 s and extension at 72 °C for 20 s. The melting curves of reactions were performed using Dissociation Curve Software (Applied Biosystems, Sparta Township, NJ, USA), and an additional 20 min was required after the program of real time PCR. The products were denatured and annealed at different temperatures to detect their suitable melting temperatures. Primer-dimers or unspecific fragments observed in samples were excluded. The threshold cycle number (*C*_t_) values of the *β-actin* gene and *ND2* gene were determined in the same quantitative PCR reaction. Each measurement was performed more than three times and normalized against a serial dilution of control DNA in each experiment. *C*_t_ values were used as measurement of the input copy number and the formula used to quantify mtDNA copy number relative to the *β-actin* gene is shown below: Relative copy number (Rc) = 2(2^Δ*C*t^), where Δ*C*_t_ is the *C*_tβ-actin_ − *C*_tND2_. The respective intra-assay coefficients of variation of *C*t were about 2.1% and 3.4% for *ND2* and *β-actin* genes, respectively.

### 4.3. Hypoxic Condition

Cells were incubated in PROOX model 110 chamber (Biospherix, Redfield, NY, USA). During incubation, a humidified environment at 37 °C, 94% N_2_, 4% CO_2_, and 1% O_2_ was maintained. Different hypoxia times ranged from 0 to 8 h were used to determine the response.

### 4.4. Western Blot Analysis

The cells were plated at a density of 2 × 10^6^ cells per well in 6-cm plates (Nunc, Roskilde, Denmark). After treatment in hypoxic camber, the cells were lysed with buffer containing 150 mM NaCl, 50 mM HEPES pH 7, 1% Triton X-100, 10% glycerol, 1.5 mM MgCl_2_, 1 mM EGTA, and protease inhibitor, then harvested to isolate protein extract. The proteins were separated via SDS-PAGE by using an 8–15% polyacrylamide gel, and then transferred onto polyvinylidene fluoride (PVDF) membrane (Millipore). The membrane was blocked using SuperBlock Blocking Buffer (Thermo Scientific, Waltham, MA, USA) for 0.5 h at room temperature, and then incubated overnight with primary antibodies at 4 °C. Following incubation of the secondary antibody with HRP for 60 min at room temperature, the signals on the membrane were detected using ECL-plus luminal solution (Pierce, Rockford, IL, USA) and X-ray film. The primary antibodies used were as follows: HIF-1α antibody (Cell Signaling Technology, Danvers, MA, USA); Pyruvate Dehydrogenase antibody (PDH, Cell Signaling Technology); Pyruvate Dehydrogenase kinase 1 antibody (PDK1, Cell Signaling Technology); anti-lactic dehydrogenase-A antibody (LDH-A, Abcam Biotech, Cambridge, UK); Dynamin-related protein antibody (DRP1, abcam Abcam Biotech, Cambridge, UK); Fission-1 antibody (FIS1, Abcam Biotech, Cambridge, UK); Mitofusin-1 antibody (MFN1, Abcam Biotech, Cambridge, UK); Mitofusin-2 antibody (MFN2, Abcam Biotech, Cambridge, UK); Optic atrophy-1 antibody (OPA1, Millipore Cor., Billerica, MA, USA); Peroxisome proliferator-activated receptor γ coactivator 1α antibody (PGC-1α, Millipore Cor., Billerica, MA, USA); mitochondrial transcription factor A antibody (TFAM, Santa Cruz Biotechnology Inc., Dallas, TX, USA); and β-actin (Millipore Cor., Billerica, MA, USA). The β-actin expression was used as the internal control.

### 4.5. Determenting of Cellular Reactive Oxygen Species (ROS)

The oxidation products in the cells were evaluated by measuring the levels of hydrogen peroxide (H_2_O_2_) using 2′,7′-dichlorofluorescin diacetate (DCFH-DA; Sigma, St. Louis, MO, USA). This probe is accumulated by cells and hydrolyzed by the cytoplasmic esterase to 2′,7′-dichlorofluorescin (DCFH), which on reaction with ROS provides the fluorescent product 2′,7′-dichlorofluorescein (DCF) which can be detected at an emission wavelength of 530 nm (excitation wavelength of 485 nm). Thus, the cells were plated at a concentration of 4 × 10^5^ cells per well in 6-well plates (Nunc, Roskilde, Denmark). After treatment in hypoxic chamber, DCFH-DA was added at a final concentration of 50 µg/ml in culture medium. Following incubation for 30 min at 37 °C, the cells were harvested and washed twice with PBS and finally added into 1 ml PBS. Finally, FACSCalibur flow cytometer (BD Biosciences, San Jose, CA, USA) was used to detect cellular ROS and the result was analyzed using the flow cytometry analysis software BD CellQuest (Becton Dickinson, San Jose, CA, USA).

### 4.6. Measurement of Mitochondrial Reactive Oxygen Species (ROS)

The levels of mitochondrial superoxide (O_2_^−^) produced in the cells were quantified by using a MitoSOX Red kit (Molecular probe, Invitrogen), which contains a redox-sensitive dye with hydroethidine linked by a hexyl carbon chain to a triphenylphosphonium group, used to target the mitochondrial matrix due to the negative membrane potential across the inner membrane of mitochondria. The cells were plated 4 × 10^5^ cells per well in 6-well plates (Nunc, Roskilde, Denmark). After treatment in hypoxic chamber, MitoSOX Red was added at a final concentration of 1.0 µM in HBSS (Gibco, Carlsbad, CA, USA). The cells were then incubated for 10 min at 37 °C, and then they were harvested and washed twice with PBS and finally added into 1 mL PBS. Identically, mitochondrial ROS was measured using FACSCalibur flow cytometer (BD Biosciences, San Jose, CA, USA) and analyzed using the flow cytometry analysis software BD CellQuest (Becton Dickinson, San Jose, CA, USA).

### 4.7. Measurement of Mitochondrial Membrane Potential

Rhodamine 123 (Molecular Probes, Eugene, OR, USA) was used to measure quantitatively mitochondrial membrane potential (Δψ_m_). The rate of fluorescence quenching is proportional to the mitochondrial membrane potential. Cells were exposed to hypoxia (1% O_2_, 5% CO_2_, 95% N_2_) for 4 h. Cells incubated in normoxic conditions (5% CO_2_/95% air) were controls. In total, 1 × 10^6^ live cells were stained with 0.5 µM rhodamine 123 for 30 min, 37 °C and then washed with ice cold PBS twice to completely remove Rhodamine from the medium. Ten thousand cells were acquired and analyzed by flow cytometry in each sample. A FACSCalibur flow cytometer (BD Biosciences, San Jose, CA, USA) was used. The data was analyzed later with using the flow cytometry analysis software BD CellQuest (Becton Dickinson, San Jose, CA, USA), by gated 10^2^–10^4^ area.

### 4.8. RNAiMAX-Mediated Transfection of SK-N-AS and ρ^0^ Cells with siRNA

SK-N-AS and ρ^0^ cell were seeding in triplicate at a density of 5 × 10^5^ cells per well in 6-cm dish. Cells were transfected with non-targeting siRNA (Santa Cruz, CA, USA), DRP1 siRNA (10 nM) (Santa Cruz, CA, USA) and HIF-1α siRNA (20 nM) (Santa Cruz, CA, USA) in 50 µL of Opti-MEM medium after overnight incubation. Then, 8 µL of RNAiMAX (Invitrogen) was gently mixed with 150 µL of Opti-MEM medium and incubated for 5 min at room temperature. After the 5 min incubation, the diluted DNA with the diluted RNAiMAX was combined and incubated for another 30 min at room temperature to allow the siRNA-RNAiMAX mix (Invitrogen) complexes to form. The 100 µL siRNA-RNAiMAX mixtures of each well were mixed gently by rocking the plate and incubated at 37 °C in a CO_2_ incubator for 24 h and then change to 10% FBS medium. Following an overnight incubation, the cells were treated with and without hypoxia for 4 h.

### 4.9. Quantitative RT-PCR Analysis of HIF-1α, PGC1-α, TFAM and SSBP

After 4 h treatment in hypoxic camber, total cellular RNA were isolated from cells using Tri reagent^®^ (Zymo Research, CA, USA) according to manufacturer’s instructions. Conversion of mRNA into double-stranded cDNA was carried out by PrimeScript™ RT reagent (TaKaRa Bio Inc., Shiga, Japan). For quantifying PGC1-α, TFAM, and SSBP, the LightCycler 480 System (Roche) was be used. SYBR Green I master mix was used to perform reaction of PCRs. Primers for human HIF-1α, PGC1-α, TFAM, SSBP, and β-actin were as follows: HIF-1α: 5′-CAGATCTCGGCGAAGTAAAGAA-3′ and 5′-GATGGTAAGCCTCATCACAGAG-3′; PGC1-α: 5′-CGTTCAAGATCGCCCTACA-3′ and 5′-CCTCTCAGACTCTCGCT-3′; TFAM: 5′-GCTAAGGGTGATTCACCGCA-3′ and 5′-ATCCTTTCGTCCAACTTCAATC-3′; SSBP: 5′-CCTCAGAGACGTGGCATATCAA-3′ and 5′-CGCCTCACATTATTTTTATCCATGT-3′; and β actin: 5′-TCACCCACACTGTGCCCATCTACGA-3′ and 5′-CAGCGGAACCGCTCATTGCCAATGG-3′.

The condition of PCR was 10 min at 95 °C, followed by 45 cycles of denaturation at 95 °C for 10 s, annealing at 60 °C for 12 s, primer extension at 72 °C for 20 s, melting 95 °C for 5 s followed by 65 °C 1 min and cooling for 40 °C 10 s.

### 4.10. Immunofluorescence Analysis and Mitochondrial Integrity Measurement

SK-N-AS and ρ^0^ cells were plated at a density of 1 × 10^4^ cells per well in 12-well plates (Nunc, Denmark) with a round glass cover slide. After treatment in hypoxic camber, the cells were then fixed in 4% paraformaldehyde and permeated by using a buffer containing 1% bovine serum albumin and 0.1% Triton X-100 in PBS and further incubated with primary antibodies for 2 h and nuclear staining with DAPI, followed by incubation with Alexa 546-conjugated secondary antibody for 1 h at room temperature. The slides were observed under fluorescence microscope after mounting the cells in Fluoromount media (Sigma). Images were obtained in a darkened room with fluorescence microscope (Leica DMI3000). As for siRNA interference study, cells were co-transfected with 0.5 µg mito DsRed and siRNA. The images of fusion and fission of mitochondria were carried out using a Zeiss LSM 5 Pascal Confocal microscopy (Carl Zeiss Microimaging, Thornwood, NY, USA). To assess the integrity of mitochondria, acquired raw images were filtered into 8 bit image type, and the signals of each mitochondrial images were consistent to the same threshold level. Finally, integrity of mitochondria was measured by using wand (tracing) tool of ImageJ (Betheda, MD, USA).

### 4.11. Statistical Analysis

The result was expressed as mean ± SD. Multiple comparisons were carried out by *t*-test or one-way analysis of variance in SPSS 11.5 for Windows (Chicago, IL, USA). *p*-Values < 0.05 were considered significant.

## 5. Conclusions

Our study demonstrates that mitochondrial fission and related proteins are up-regulated by HIF-1α, while mtDNA participates in many downstream steps of the HIF-1α mediated pathway. The intervention of the HIF-1α related pathway can be a potential target for the treatment of hypoxic injury in central nervous system disorders, such as ischemic stroke and hypoxic encephalopathy. Further studies are warranted to evaluate its clinical utility as a novel therapeutic approach.

## Supplementary Materials

Supplementary materials can be found at www.mdpi.com/1422-0067/18/6/1220/s1.
