# Supplementary material for: mtDNA as a Mediator for Expression of Hypoxia-Inducible Factor 1α and ROS in Hypoxic Neuroblastoma Cells
Source: Int J Mol Sci. 2017 Jun 7;18(6):1220. doi: 10.3390/ijms18061220 (PMC5486043; doi:10.3390/ijms18061220)
Supplement: Supplementary file 1 [file ijms-18-01220-s001.pdf]

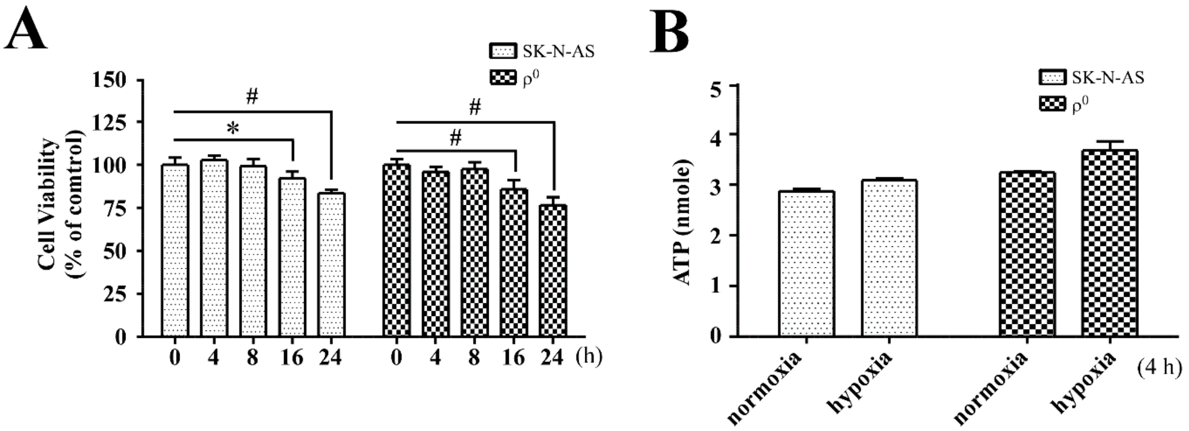

Figure S1. Functional differences between SK-N-AS and ϱ° cells. (A) The percentage of viable SK-N-AS and ϱ° cells after 0, 4, 8, 16 and 24 hours hypoxia were determined by using alarmBlue assay. (B) ATP productions of both hypoxic cells for four hours were measured with ELISA. Statistical significance (*P<0.05; #P < 0.005) was determined using the one way ANOVA compared to normoxic cells.

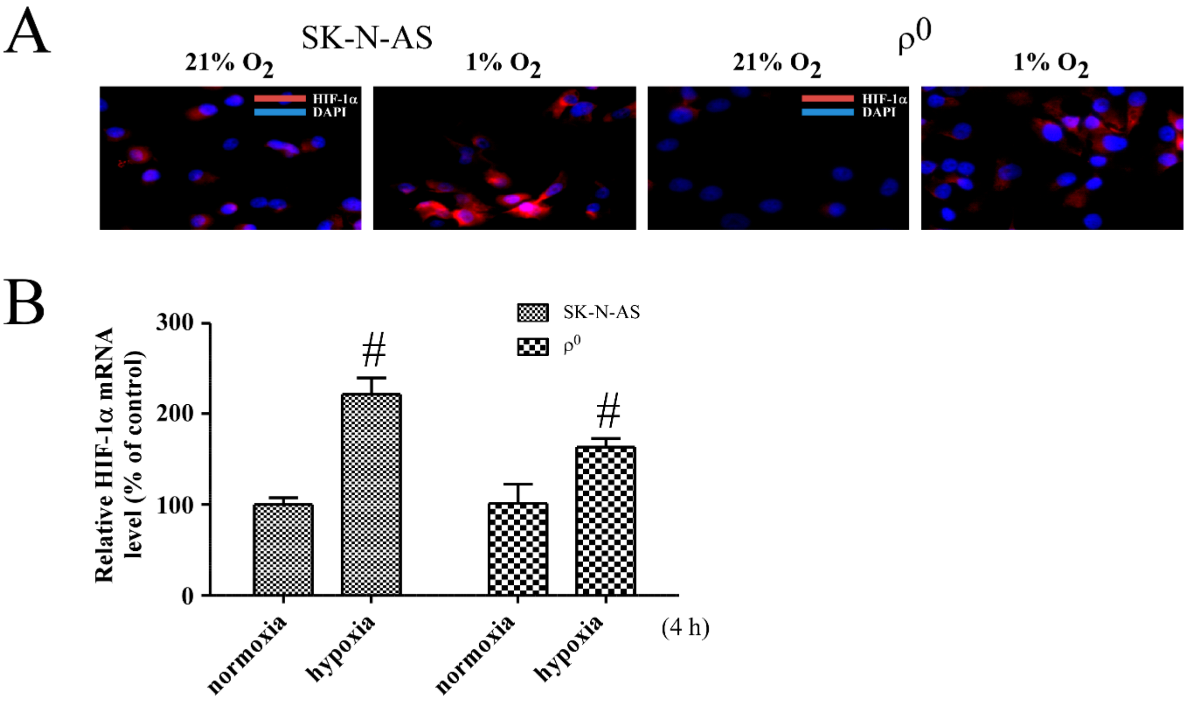

Figure S2. The expression of HIF-1α in protein and mRNA levels. (A) The imaging showed the results of immunofluorescence staining of the expression of HIF-1α (red) and DAPI (blue) after 4 hours hypoxic (1% O_2) and normoxic (21% O_2) exposure in SK-N-AS and ϱ° cells. Fluorescence images were acquired using the same acquisition parameters at ×20 magnification. (B) The mRNA expression level of HIF-1α after four hypoxic hours in SK-N-AS and ϱ° cells were determined by q-PCR. # represented P<0.005 when compared to normoxic condition.

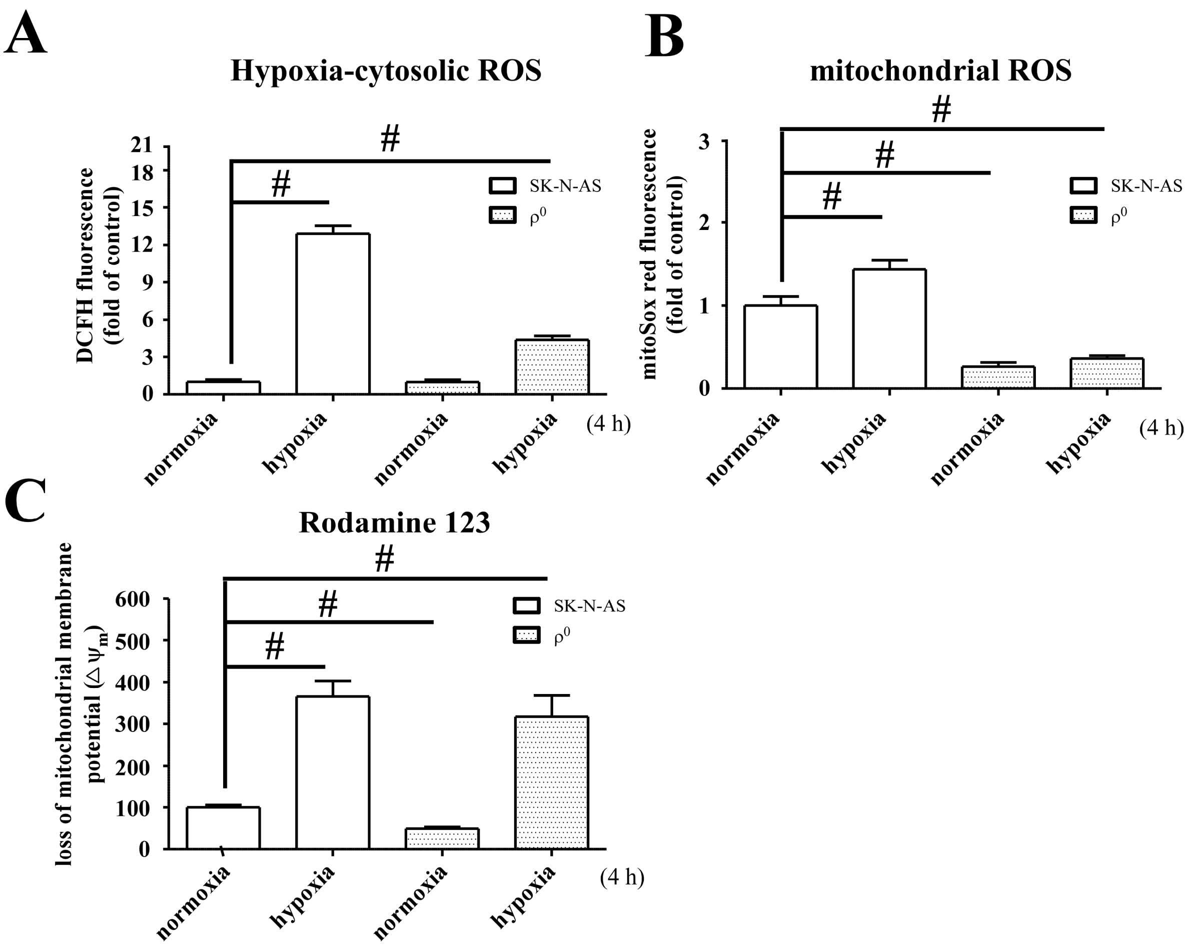

Figure S3. Hypoxia induces reactive oxygen species (ROS) and loss of mitochondrial membrane potential (ΔΨm). Intracellular H_2O_2 production (A) or mitochondrial superoxide (B) under normoxia or after hypoxia for 4 h were determined by flow cytometry with DCFH-DA or MitoSox red individually in both SK-N-AS and ϱ° cells. (C) Loss of mitochondrial membrane potential in normoxic or hypoxic SK-N-AS and ϱ° cells were measured by Rodamine 123. The percentage loss of ΔΨm indicated the number of ΔΨm collapsed cells after exposure to hypoxia. These results represent mean ± SE of more than three independent experiments. Statistical significance (#P < 0.005) was determined using the one way ANOVA compared to normoxic SK-N-AS cells.
